# Supplementary material for: Multiplex transcriptional characterizations across diverse bacterial species using cell‐free systems
Source: Mol Syst Biol. 2019 Aug 14;15(8):e8875. doi: 10.15252/msb.20198875 (PMC6692573; doi:10.15252/msb.20198875)
Supplement: Supplementary file 1 — Appendix [file MSB-15-e8875-s001.pdf]

Appendix

Multiplex transcriptional characterizations across diverse bacterial species using cell-free systems

Authors: Sung Sun Yim#, Nathan I. Johns#, Jimin Park, Antonio L.C. Gomes, Ross M. McBee, Miles Richardson, Carlotta Ronda, Sway P. Chen, David Garenne, Vincent Noireaux, Harris H. Wang*

*Correspondence to: hw2429@columbia.edu

#These authors contributed equally

This PDF file includes:

Appendix Methods

Appendix Tables S1 to S6

Appendix Figures S1 to S14

Table of Contents

Appendix Methods

- Template-switching adaptor ligation

Appendix Table S1: Bacterial species used in this study and their growth conditions
Appendix Table S2: Composition of media, buffer and components used in this study
Appendix Table S3: Composition of cell-free systems for each bacterial species
Appendix Table S4: Plasmids and libraries used in this study
Appendix Table S5: Primers used in this study
Appendix Table S6: Reproducibility between biological replicates in this study

Appendix Figure S1: DNA regulatory sequence analysis in cell-free transcription systems
Appendix Figure S2: Robustness of DRAFTS transcriptional measurements in *E. coli* cell-free expression system
Appendix Figure S3: Transcription start site and motif analysis of library regulatory sequences
Appendix Figure S4: Transcriptional and translational yields from diverse cell-free expression systems
Appendix Figure S5: Comparison of *in vitro* and *in vivo* transcriptional measurements in 7 bacterial species
Appendix Figure S6: Examination of library DNA abundances identifies sequences subject to restriction enzyme digest in *Lactococcus lactis*
Appendix Figure S7: Comparisons of RS1383 transcriptional profiles across 10 bacterial species using DRAFTS
Appendix Figure S8: RNA polymerase subunit sigma70 as an alternative metric of evolutionary divergence
Appendix Figure S9: Functional category of regulatory sequence gene origin does not influence activity
Appendix Figure S10: Error distribution of linear regression models for transcription activation in 10 bacterial species
Appendix Figure S11: Evaluation of linear regression models on a separate dataset
Appendix Figure S12: Transcriptional activities from dual-species hybrid lysates
Appendix Figure S13: Translational activities from dual-species hybrid lysates
Appendix Figure S14: Hybrid lysates alleviate species-selectivity of promoters

Appendix Methods

Template-switching adaptor ligation

Data in **Appendix Figure S5** was prepared by Template-switching, an alternative method for addition of common adaptor sequences to cDNA using the following reverse transcription reaction:

5 μL	Total RNA sample (up to 5 μg)
1 μL	Reverse transcription primer (20 μM)
1 μL	10 mM dNTPs (Invitrogen)
6.5 μL	Nuclease free water

Components were incubated at 65 ºC for 5 minutes and chilled on ice for 1 minute. The following were then added to the reaction:

4 μL	5x RT buffer
0.5 μL	RNase inhibitor (Ribolock Thermo Scientific)
1 μL	Maxima reverse transcriptase (Thermo Scientific)
1 μL	Template switching oligo (20 μM)

The reverse transcription reaction was carried out as follows on a 96-well thermocycler (BioRad):

42ºC for 90 minutes
50ºC for 2 minutes
42ºC for 2 minutes
Repeat the two steps above for 9 cycles
85ºC for 5 minutes
4ºC hold

The completed reaction was incubated with 1 μL RNase H at 37ºC for 30 minutes.

Appendix Table S1. Bacterial species used in this study and their growth conditions

Species	Strain (Source)	Medium	Temp.	Aeration
E. coli	BL21	2xYT+P	37℃	Shaking (220 rpm)
E. fergusonii	Isolate (Murine feces)	2xYT+P	37℃	Shaking (220 rpm)
S. enterica	Serovar Typhi Ty2	2xYT+P	37℃	Shaking (220 rpm)
K. oxytoca	M5A1 (DSM 7342)	2xYT+P	30℃	Shaking (220 rpm)
P. agglomerans	Isolate (Agricultural waste)	2xYT+P	30℃	Shaking (220 rpm)
P. putida	KT2440 (ATCC 47054)	2xYT+P	30℃	Shaking (220 rpm)
V. natriegens	ATCC 14048	BHI+v2 salt	37℃	Shaking (220 rpm)
B. subtilis	BD3182 (168 derivative)	2xYT+P	30℃	Shaking (220 rpm)
C. glutamicum	ATCC 13032	BHI	30℃	Shaking (220 rpm)
L. lactis	ATCC 11454	MRS	37℃	Static

Appendix Table S2. Composition of media, buffer and components used in this study

Medium	Composition
2xYT+P	10 g/L Yeast extract (BD, 288620), 16 g/L Tryptone (BD, 211705), 5 g/L NaCl, 40 mM Potassium phosphate dibasic, 22 mM Potassium phosphate monobasic
BHI	37 g/L BHI (Brain Heart Infusion) (BD, 237500)
BHI+v2 salts	37 g/L BHI (Brain Heart Infusion) (BD, 237500) + v2 salts (204 mM NaCl, 4.2 mM KCl, 23.14 mM $MgCl_2$)*
MRS	55 g/L MRS (BD, 288130)

*[Weinstock et al., 2016. *Nat Methods* **13**, 849-51]

Buffer	Composition
S30A*	14 mM Mg-glutamate, 60 mM K-glutamate, 50 mM Tris base (pH 7.7 - titrate with acetic acid) + supplement 1 M DTT sol to make 2 mM DTT concentration before use
S30B	14 mM Mg-glutamate, 60 mM K-glutamate, 5 mM Tris base (pH 8.2 - titrate with 2 M Tris base solution) + supplement 1 M DTT sol to make 1 mM DTT concentration before use

*[Sun et al., 2013. *J Vis Exp* **79**, e50762]

Components	Composition
Amino acids*	RTS Amino Acid Sampler kit (6 mM of each amino acid, except leucine which is 5 mM)
Energy solution (PGA)*	700 mM HEPES (pH 8), 21 mM ATP, 21 mM GTP, 12.6 mM CTP, 12.6 mM UTP, 2.8 mg/mL tRNA, 3.64 mM CoA, 4.62 mM NAD, 10.5 mM cAMP, 0.95 mM Folinic acid, 14 mM Spermidine + 420 mM 3-PGA
Energy solution (PEP)	700 mM HEPES (pH 8), 21 mM ATP, 21 mM GTP, 12.6 mM CTP, 12.6 mM UTP, 2.8 mg/mL tRNA, 3.64 mM CoA, 4.62 mM NAD, 10.5 mM cAMP, 0.95 mM Folinic acid, 14 mM Spermidine + 420 mM PEP

*[Sun et al., 2013. *J Vis Exp* **79**, e50762]

Appendix Table S3. Composition of cell-free systems for each bacterial species

E. coli

Component	Stock conc.	Final conc.	Volume*
Lysate**	1	0.33**	28.5 µL
Mg-glutamate	500 mM	9	1.54 µL
K-glutamate	3 M	30	0.86 µL
Amino Acids	6 mM each***	1.5 mM each	21.38 µL
Energy Solution (PGA)	14x	1x	6.11 µL
PEG-8000	40%	2%	4.28 µL
Water			1.47 µL

E. fergusonii

Component	Stock conc.	Final conc.	Volume*
Lysate**	1	0.3**	25.71 µL
Mg-glutamate	500 mM	9	1.54 µL
K-glutamate	3 M	30	0.86 µL
Amino Acids	6 mM each***	1.5 mM each	21.38 µL
Energy Solution (PGA)	14x	1x	6.11 µL
PEG-8000	40%	2%	4.28 µL
Water			4.26 µL

S. enterica

Component	Stock conc.	Final conc.	Volume*
Lysate**	1	0.33**	28.17 µL
Mg-glutamate	500 mM	6	1.03 µL
K-glutamate	3 M	30	0.86 µL
Amino Acids	6 mM each***	1.5 mM each	21.38 µL
Energy Solution (PGA)	14x	1x	6.11 µL
PEG-8000	40%	2%	4.28 µL
Water			2.32 µL

*enough for ~8 single reactions

**input as volumetric ratio decided based on total protein conc. of the lysate*

***except Leucine which is 5 mM

Appendix Table S3. (Continued)

K. oxytoca

Component	Stock conc.	Final conc.	Volume*
Lysate**	1	0.3**	25.79 µL
Mg-glutamate	500 mM	9	1.54 µL
K-glutamate	3 M	90	2.57 µL
Amino Acids	6 mM each***	1.5 mM each	21.38 µL
Energy Solution (PGA)	14x	1x	6.11 µL
PEG-8000	40%	2%	4.28 µL
Water			2.47 µL

P. agglomerans

Component	Stock conc.	Final conc.	Volume*
Lysate**	1	0.28**	23.52 µL
Mg-glutamate	500 mM	6	1.03 µL
K-glutamate	3 M	90	2.57 µL
Amino Acids	6 mM each***	1.5 mM each	21.38 µL
Energy Solution (PGA)	14x	1x	6.11 µL
PEG-8000	40%	2%	4.28 µL
Water			5.26 µL

P. putida

Component	Stock conc.	Final conc.	Volume*
Lysate**	1	0.33**	28.5 µL
Mg-glutamate	500 mM	9	1.54 µL
K-glutamate	3 M	30	0.86 µL
Amino Acids	6 mM each***	1.5 mM each	21.38 µL
Energy Solution (PGA)	14x	1x	6.11 µL
PEG-8000	40%	2%	4.28 µL
Water			1.47 µL

*enough for ~8 single reactions

**input as volumetric ratio decided based on total protein conc. of the lysate

***except Leucine which is 5 mM

Appendix Table S3. (Continued)

V. natriegens

Component	Stock conc.	Final conc.	Volume*
Lysate**	1	0.31**	26.23 µL
Mg-glutamate	500 mM	6	1.03 µL
K-glutamate	3 M	90	2.57 µL
Amino Acids	6 mM each***	1.5 mM each	21.38 µL
Energy Solution (PGA)	14x	1x	6.11 µL
PEG-8000	40%	2%	4.28 µL
Water			2.55 µL

B. subtilis

Component	Stock conc.	Final conc.	Volume*
Lysate**	1	0.32**	27.67 µL
Mg-glutamate	500 mM	9	1.54 µL
K-glutamate	3 M	0	0 µL
Amino Acids	6 mM each***	1.5 mM each	21.38 µL
Energy Solution (PGA)	14x	1x	6.11 µL
PEG-8000	40%	2%	4.28 µL
Water			3.16 µL

C. glutamicum

Component	Stock conc.	Final conc.	Volume*
Lysate**	1	0.33**	28.5 µL
Mg-glutamate	500 mM	9	1.54 µL
K-glutamate	3 M	90	2.57 µL
Amino Acids	6 mM each***	1.5 mM each	21.38 µL
Energy Solution (PGA)	14x	1x	6.11 µL
PEG-8000	40%	2%	4.28 µL
Water			0 µL

*enough for ~8 single reactions

**input as volumetric ratio decided based on total protein conc. of the lysate

***except Leucine which is 5 mM

Appendix Table S3. (Continued)

L. lactis

Component	Stock conc.	Final conc.	Volume*
Lysate**	1	0.33**	28.5 µL
Mg-glutamate	500 mM	6	1.03 µL
K-glutamate	3 M	0	0 µL
Amino Acids	6 mM each***	1.5 mM each	21.38 µL
Energy Solution (PEP)	14x	1x	6.11 µL
PEG-8000	40%	2%	4.28 µL
Water			2.84 µL

*enough for ~8 single reactions

**input as volumetric ratio decided based on total protein conc. of the lysate

***except Leucine which is 5 mM

Appendix Table S4. Plasmids and libraries used in this study

Plasmid	Origin	Antibiotic	Description
pTOPO-F30-Broccoli	pBR322	Carbenicillin	P_{Gen_18145}-F30-Broccoli*
pTXTL-P70a-deGFP	pBR322	Carbenicillin	P_{70a}-deGFP**

*[Filonov et al., 2014. *J Am Chem Soc* **136**, 16299-308]

[Sun et al., 2013. *J Vis Exp* **79, e50762]

Library	Backbone	Origin	Antibiotic	Related Figures
RS234	pNJ1	p15A	Carbenicillin	Fig. 1B, 1C,<br>Fig. S2A, C, D, E, Fig. S7
RS29249	pNJ1	p15A	Carbenicillin	Fig. 1D, 1E,<br>Fig. S2B, Fig. S3
RS1383	pNJ1	p15A	Carbenicillin	Fig. 3, Fig. S1,<br>Fig. S8, Fig. S9, Fig. S10,
RS7003	pNJ1	p15A	Carbenicillin	Fig. 4, Fig. 5
RS234 for Ko, Vn	pNJ7	p15A	Chloramphenicol	Fig. S7
RS234 for Pp	pNJ3	pBBR1	Carbenicillin	Fig. S7
RS234 for Bs	pNJ2	integrative,<br>pBR322	Chloramphenicol	Fig. S7
RS234 for Cg	pNJ8	pCG1,<br>p15A	Kanamycin	Fig. S7

Appendix Table S5. Primers used in this study

Primers	Description	Sequence (5'-3')
GFP-RT	Reverse transcription from GFP	TCAGATAGTGATTGTCTGG
mCherry-RT	Reverse transcription from mCherry	TACATCCCACAACGAAG
3'-Adaptor*	Adaptor for 3'-cDNA end ligation	/5Phos/NNATGTACTCTGCGTTGATACCACTGCTT/3SpC3/
5'-TSO**	Template switching oligo	AAGCAGTGGTATCAACGCAGAGTACATrGrGrG
DNA-amp1-F	Fwd primer for 1st amplification of DNA	GAGTTCAGACGTGTGCTCTTCCGATCTCGTCTAAGAAACCATTATTATCATGACA
RNA-amp1-F	Fwd primer for 1st amplification of RNA	GAGTTCAGACGTGTGCTCTTCCGATCTAAGCAGTGGTATCAACGC
Amp1-R***	Rev primer for 1st amplification of DNA and RNA	CCTACACGACGCTCTTCCGATCT[NNN]ACAGCTCTTCGCCTTTACG
Amp2-F****	Fwd primer for 2nd amplification	CAAGCAGAAGACGGCATACGAGAT[NNNNNN]GTGACTGGAGTTCAGACGTGTGCTCTTC
Amp2-R	Rev primer for 2nd amplification	AATGATACGGCGACCACCGAGATCTACACTCTTTCCCTACACGACGCTCTTCCGATCT

*/5Phos/: 5'-phosphorylation, /3SpC3/: 3'-spacer C3

**rN: RNA bases

***[NNN]: with variable lengths (NNN, NNNN, NNNNN, NNNNNN) for staggering

****[NNNNNN]: index for each sample

Appendix Table S6. Reproducibility between biological replicates in this study

Figure	Sample	Library	Pearson's r between biological replicates
**Figure 1B**	DRAFTS *E. coli* batch 1	RS234	0.9745
**Figure 1B**	DRAFTS *E. coli* batch 2	RS234	0.9891
**Figure 1D**	*E. coli in vivo*	RS29249	0.8932
**Figure 1D**	DRAFTS *E. coli*	RS29249	0.9333
**Figure 3**	DRAFTS *E. coli*	RS1383	0.955
**Figure 3**	DRAFTS *E. fergusonii*	RS1383	0.9737
**Figure 3**	DRAFTS *S. enterica*	RS1383	0.8216
**Figure 3**	DRAFTS *K. oxytoca*	RS1383	0.9357
**Figure 3**	DRAFTS *P. agglomerans*	RS1383	0.9614
**Figure 3**	DRAFTS *P. putida*	RS1383	0.9338
**Figure 3**	DRAFTS *V. natriegens*	RS1383	0.9541
**Figure 3**	DRAFTS *B. subtilis*	RS1383	0.9688
**Figure 3**	DRAFTS *C. glutamicum*	RS1383	0.9886
**Figure 3**	DRAFTS *L. lactis*	RS1383	0.9644
**Figure 4**	DRAFTS *E. coli*	RS7003	0.9621
**Figure 4**	DRAFTS *E. fergusonii*	RS7003	0.9621
**Figure 4**	DRAFTS *S. enterica*	RS7003	0.7708
**Figure 4**	DRAFTS *K. oxytoca*	RS7003	0.9575
**Figure 4**	DRAFTS *P. agglomerans*	RS7003	0.9581
**Figure 4**	DRAFTS *P. putida*	RS7003	0.9434
**Figure 4**	DRAFTS *V. natriegens*	RS7003	0.841
**Figure 4**	DRAFTS *B. subtilis*	RS7003	0.9278
**Figure 4**	DRAFTS *C. glutamicum*	RS7003	0.9795
**Figure 4**	DRAFTS *L. lactis*	RS7003	0.8323
**Figure 5**	DRAFTS *EcBs* hybrid (1:0)	RS7003	0.851
**Figure 5**	DRAFTS *EcBs* hybrid (4:1)	RS7003	0.9082
**Figure 5**	DRAFTS *EcBs* hybrid (1:4)	RS7003	0.9614
**Figure 5**	DRAFTS *EcBs* hybrid (0:1)	RS7003	0.8939
**Figure 5**	DRAFTS *EcCg* hybrid (1:0)	RS7003	0.851
**Figure 5**	DRAFTS *EcCg* hybrid (4:1)	RS7003	0.8659
**Figure 5**	DRAFTS *EcCg* hybrid (1:4)	RS7003	0.9105
**Figure 5**	DRAFTS *EcCg* hybrid (0:1)	RS7003	0.9575

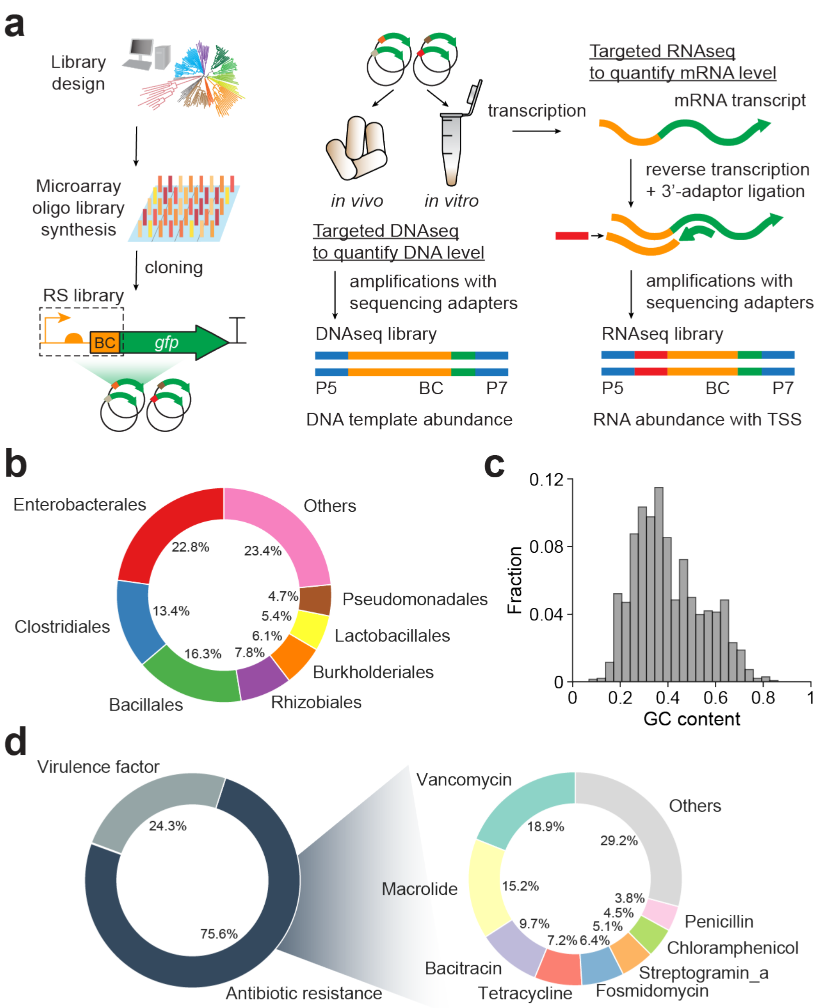

Appendix Figure S1. DNA regulatory sequence analysis in cell-free transcription systems. (a) Microarray synthesis, library cloning, and high-throughput characterization of regulatory sequences using targeted sequencing of RNA and DNA. **(b)** Phylogenetic origins, **(c)** GC content distribution, **(d)** functional categories of genes where the sequences mined from and related antibiotics of RS1383 library.

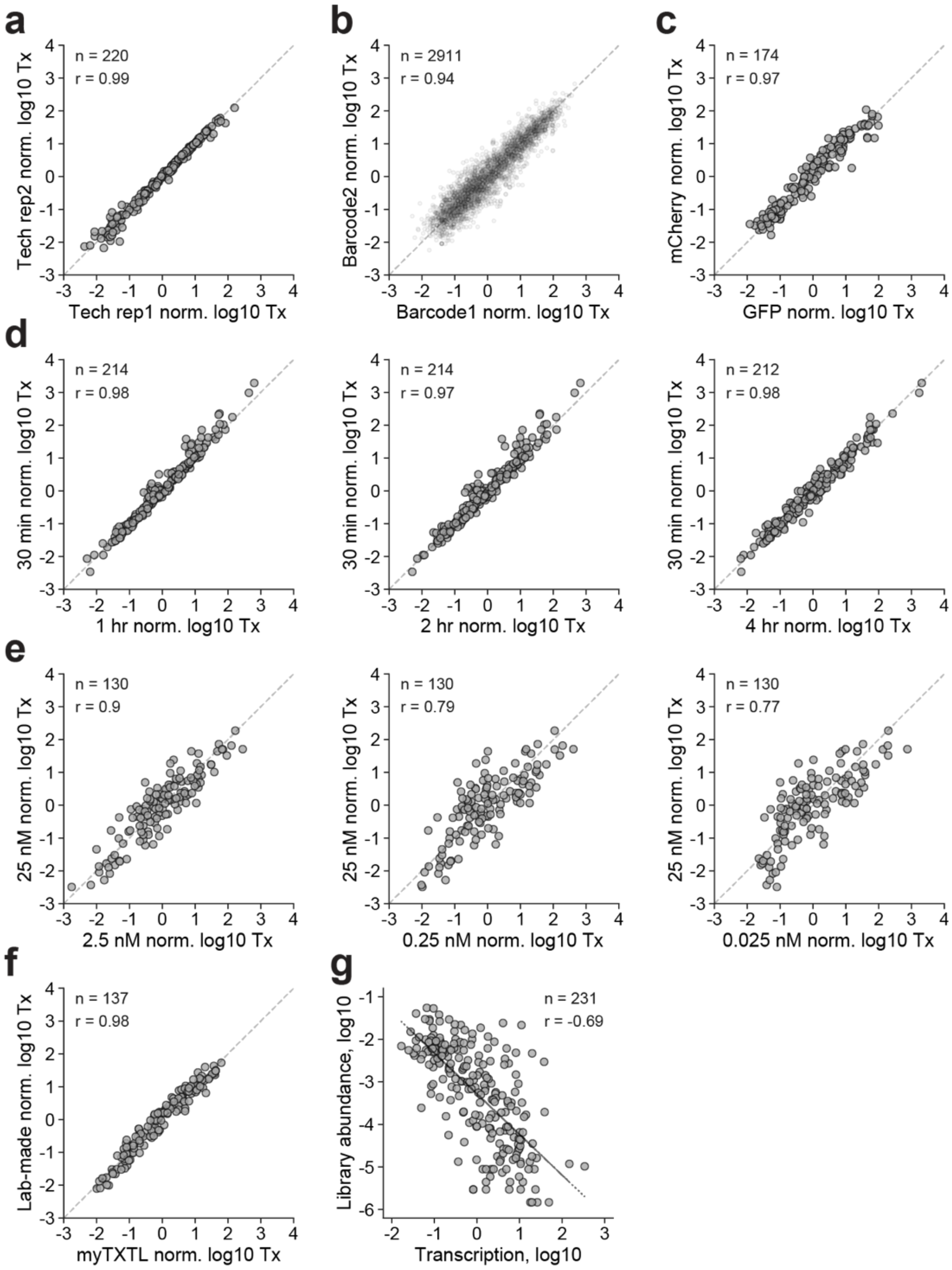

Appendix Figure S2. Robustness of DRAFTS transcriptional measurements in *E. coli* cell-free expression system. Comparison of transcriptional profiles (Tx) of regulatory sequences across **(a)** technical replicates, **(b)** alternate barcodes, **(c)** alternate reporter genes, **(d)** reaction times, **(e)** input DNA concentrations, and **(f)** commercial (myTXTL, Arbor Bioscience) and lab-made lysates. **(g)** Negative correlation between transcriptional activities and abundance of library constructs due to impact of gene expression upon cell fitness *in vivo*. Dashed lines represent y=x in **(a)**-**(f)** and linear regression in **(g)**. Sample sizes (n) and Pearson correlation coefficients (r) can be found in each plot. For normalization, transcription levels in log_{10} scale were transformed to Z-score. All measurements except **(a)** are based on merged counts from two technical replicates.

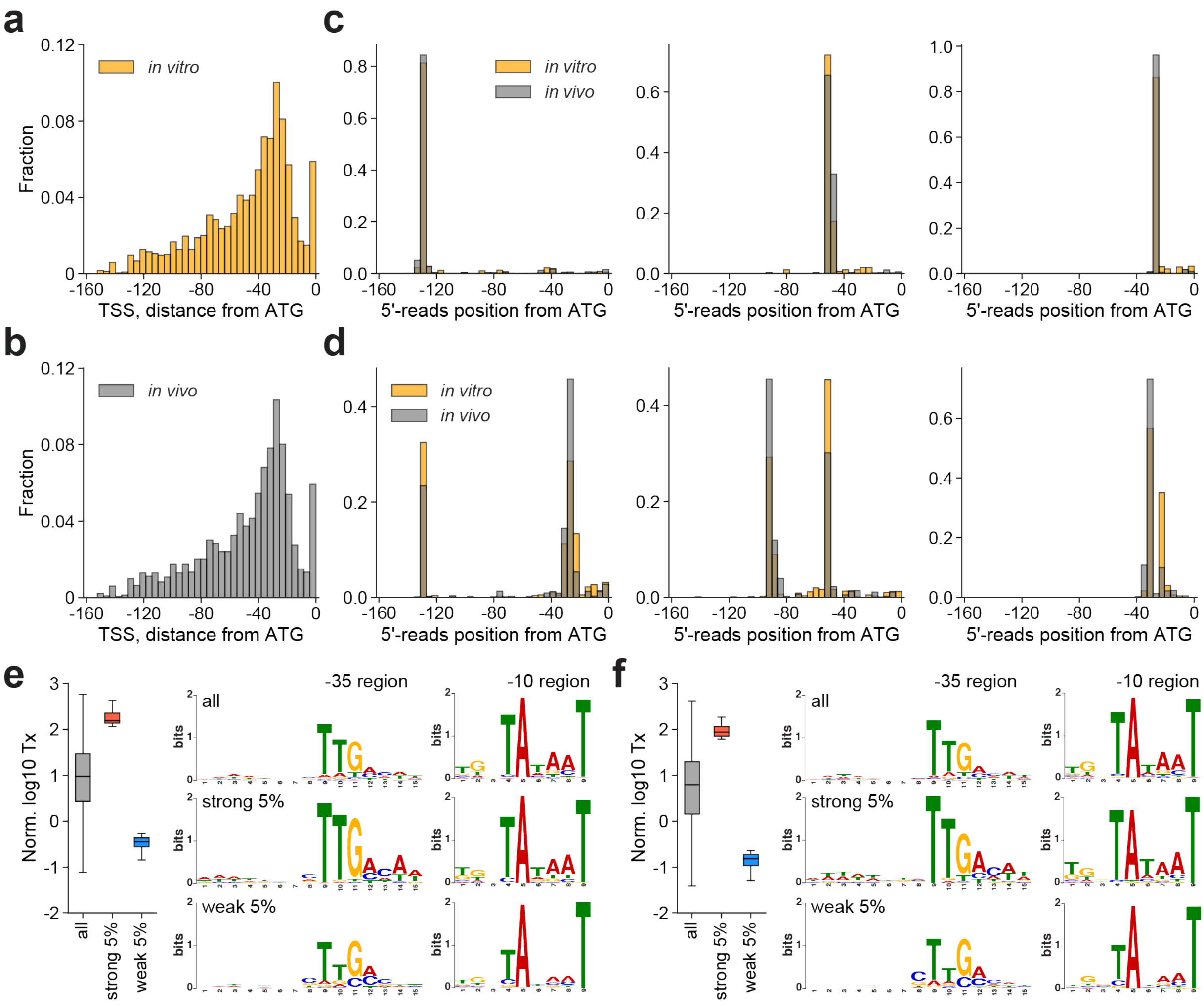

Appendix Figure S3. Transcription start site and motif analysis of library regulatory sequences. Primary TSS distributions **(a)** *in vitro* and **(b)** *in vivo*, with example read position distributions for example regulatory sequences containing **(c)** a single and **(d)** two TSSs. Motif analysis on all, strong 5%, and weak 5% promoters from **(e)** *in vitro* or **(f)** *in vivo* measurements gives similar sigma70 motifs enriched for each promoter group. Box plots are displaying the interquartile range (IQR) with median values (black line) and whiskers extending to the highest and lowest points within 1.5x the IQR. For normalization, transcription levels in log_{10} scale were transformed to Z-score. All measurements are based on two biological replicates.

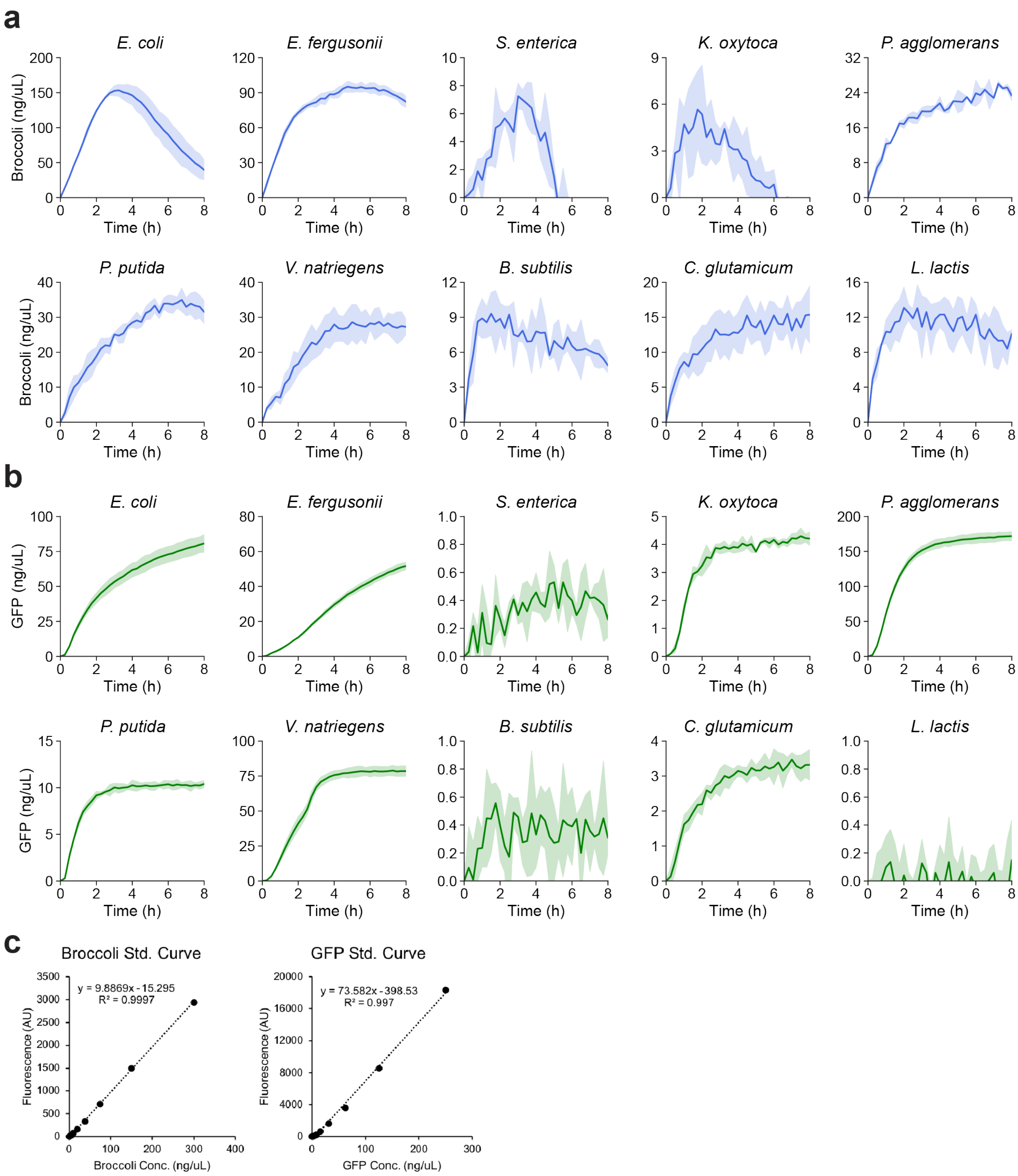

Appendix Figure S4. Transcriptional and translational yields from diverse cell-free expression systems. (a) Broccoli and **(b)** GFP expression over 8 hours. Standard curves used to quantify Broccoli and GFP in the cell-free reactions are shown in **(c)**. For Broccoli expression, 12.5 nM of pTOPO-F30-Broccoli plasmid was used as a template (50 nM for *L. lactis*). For GFP expression, 25 nM of pTXTL-P70a-deGFP plasmid was used as a template. Dashed lines represent linear regression in **(c)**. Shaded regions represent standard deviation of three biological replicates.

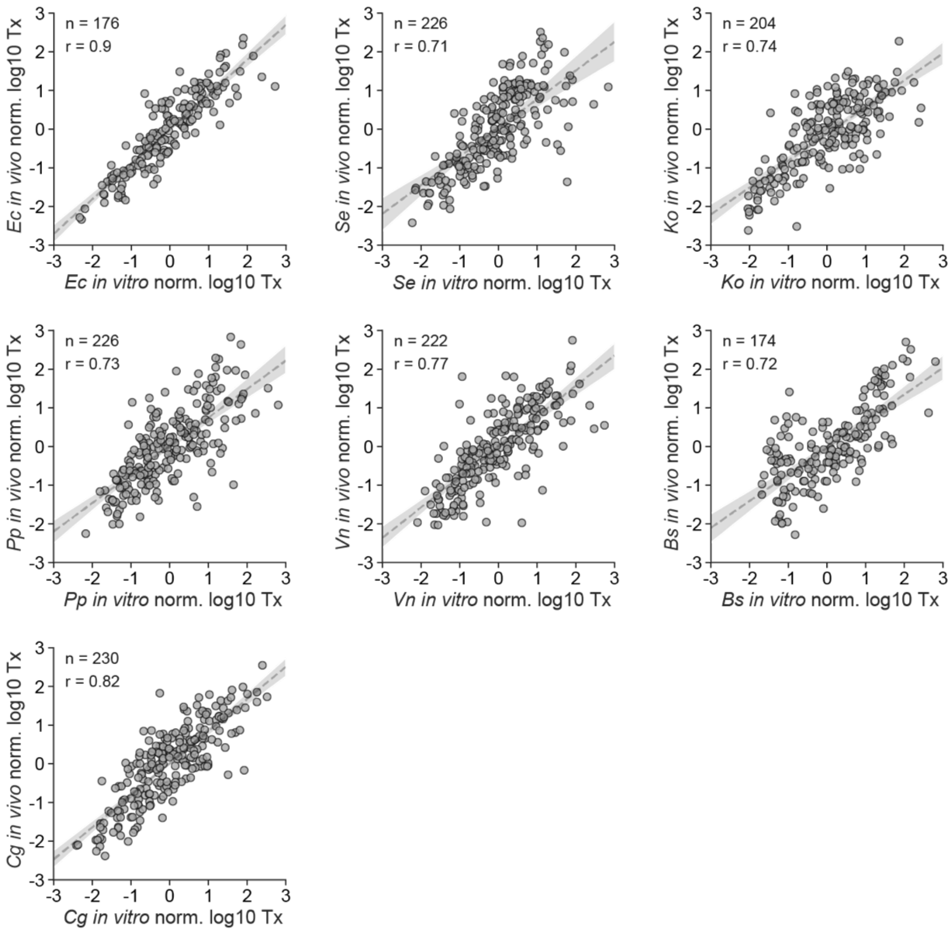

Appendix Figure S5. Comparison of *in vitro* and *in vivo* transcriptional measurements in 7 bacterial species. Transcriptional profile (Tx) correlations between *in vivo* and *in vitro* measurements in *E. coli*, *S. enterica*, *K. oxytoca*, *P. putida*, *V. natriegens*, *B. subtilis*, and *C. glutamicum*. All transcriptional measurements were made using template-switching adaptor ligation. Shaded regions represent 95% confidence interval for linear regression (dashed lines). Sample sizes (n) and Pearson correlation coefficients (r) can be found in each plot. For normalization, transcription levels in log_{10} scale were transformed to Z-score. All measurements are based on two biological replicates.

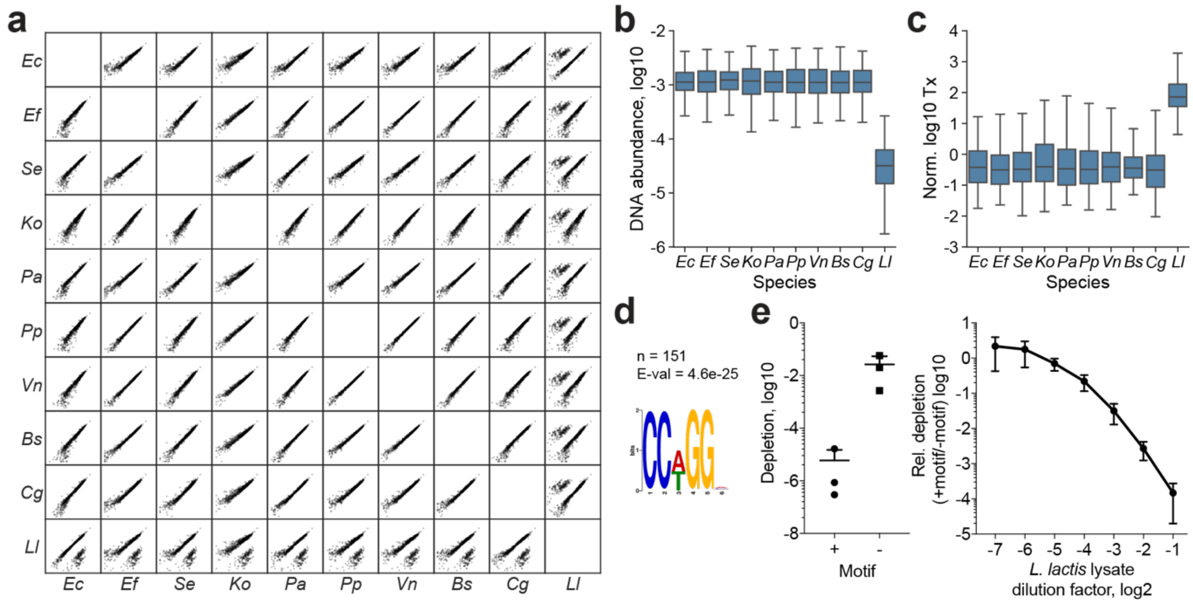

Appendix Figure S6. Examination of library DNA abundances identifies sequences subject to restriction enzyme digest in *Lactococcus lactis*. (a) Correlation of construct abundances from DNA amplicon sequences after 30-minute incubation in cell lysates from 10 species. **(b)** Box plots of DNA abundance distributions and **(c)** artificially inflated transcriptional activity (Tx) calculations for 182 regulatory sequences that are depleted by >2-fold in *L. lactis*. Box plots are displaying the interquartile range (IQR) with median values (black line) and whiskers extending to the highest and lowest points within 1.5x the IQR. **(d)** An enriched motif in *L. lactis* depleted sequences with the number of sequences (n) and motif E-value from MEME listed above the logo. The CCNGG motif had the most hits (151 sites from 182 sequences, 83%) and corresponded to a restriction enzyme (ScrFIR) found in *L. lactis* genome. **(e)** ScrFIR assay results in *L. lactis* lysate. ~200-bp linear DNA templates with/without "CCTGG" target motif sequence of ScrFIR were incubated in *L. lactis* lysates for 10 min, then depletion of the DNA templates was measured by qPCR. ~10^4-fold stronger depletion was observed from the DNA template with "CCTGG" motif sequence, and the relative depletion rates became lower as the DNA templates were incubated in more diluted lysates. For normalization, transcription levels in $\log_{10}$ scale were transformed to Z-score. All measurements are based on two biological replicates.

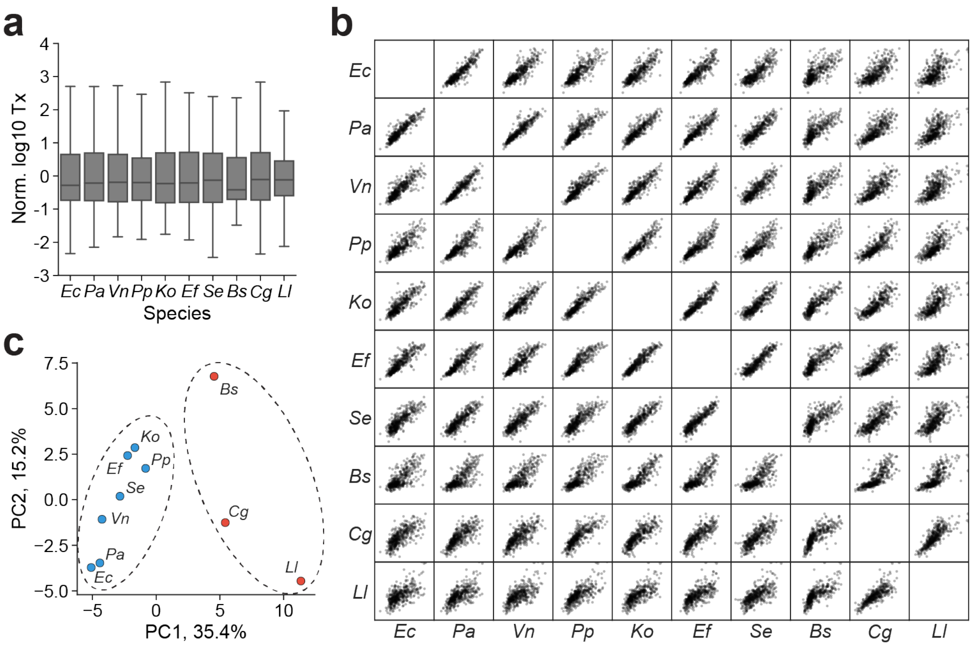

Appendix Figure S7. Comparisons of RS1383 transcriptional profiles across 10 bacterial species using DRAFTS. (a) Box plot of transcriptional activity (Tx) distributions for each species. Box plots are displaying the interquartile range (IQR) with median values (black line) and whiskers extending to the highest and lowest points within 1.5x the IQR. **(b)** Scatter plot matrix of *in vitro* transcriptional activity comparisons for 10 species. **(c)** Principal components analysis of transcriptional profile similarity for 10 species. For normalization, transcription levels in log_{10} scale were transformed to Z-score. All measurements are based on two biological replicates.

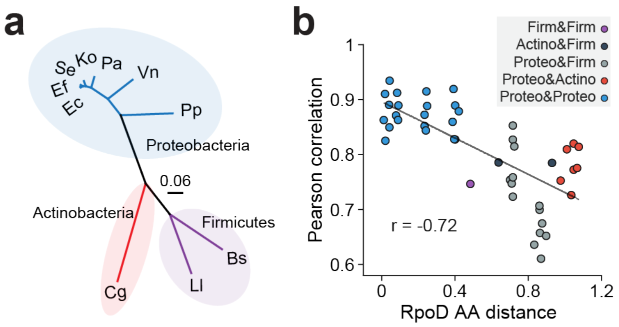

Appendix Figure S8. RNA polymerase subunit sigma70 as an alternative metric of evolutionary divergence. (a) Unrooted phylogenetic tree of 10 bacterial species used in this study based on a multiple alignment of primary sigma factor RpoD amino acid sequences using UPGMA method (distance scale of 0.06). **(b)** Correlation between amino acid sequence dissimilarity of RpoD and pairwise Pearson correlation of transcriptional profiles of 421 universally-active regulatory sequences between bacterial species.

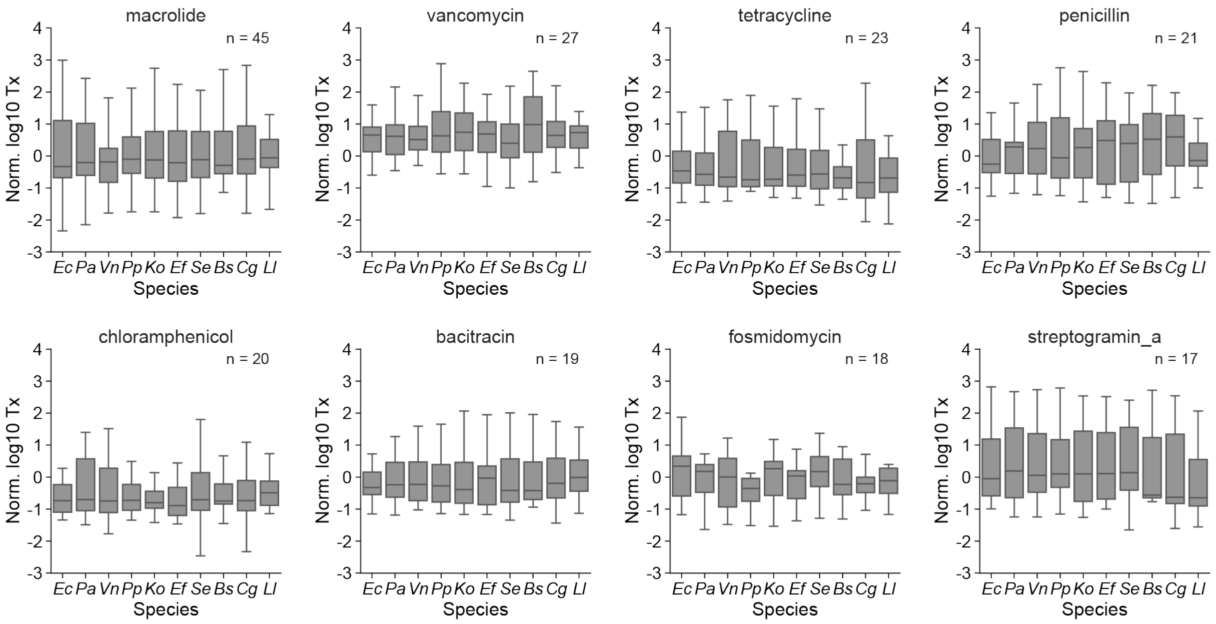

Appendix Figure S9. Functional category of regulatory sequence gene origin does not influence activity. Box plots of transcriptional activity (Tx) in 10 species for RS1383 antibiotic resistance gene regulatory sequences, grouped by resistance gene class. Box plots are displaying the interquartile range (IQR) with median values (black line) and whiskers extending to the highest and lowest points within 1.5x the IQR. For normalization, transcription levels in log_{10} scale were transformed to Z-score. All measurements are based on two biological replicates.

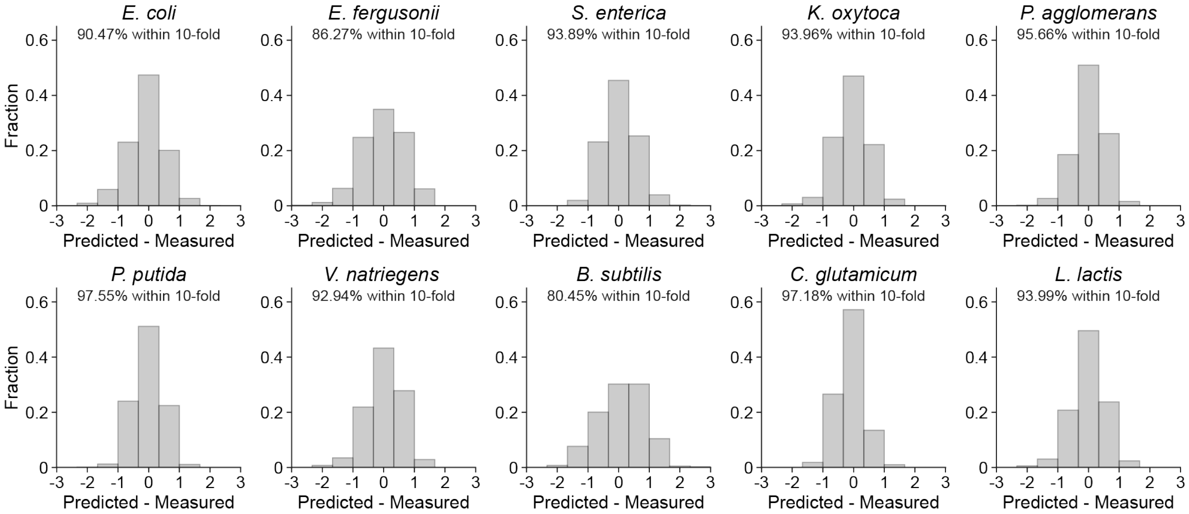

Appendix Figure S10. Error distribution of linear regression models for transcription activation in 10 bacterial species. Distributions of prediction errors (Predicted – Measured transcription activities in $\log_{10}$ scale) of linear regression models displayed in **Figure 4C**. Proportion of prediction that are within 10-fold error window is shown in each plot.

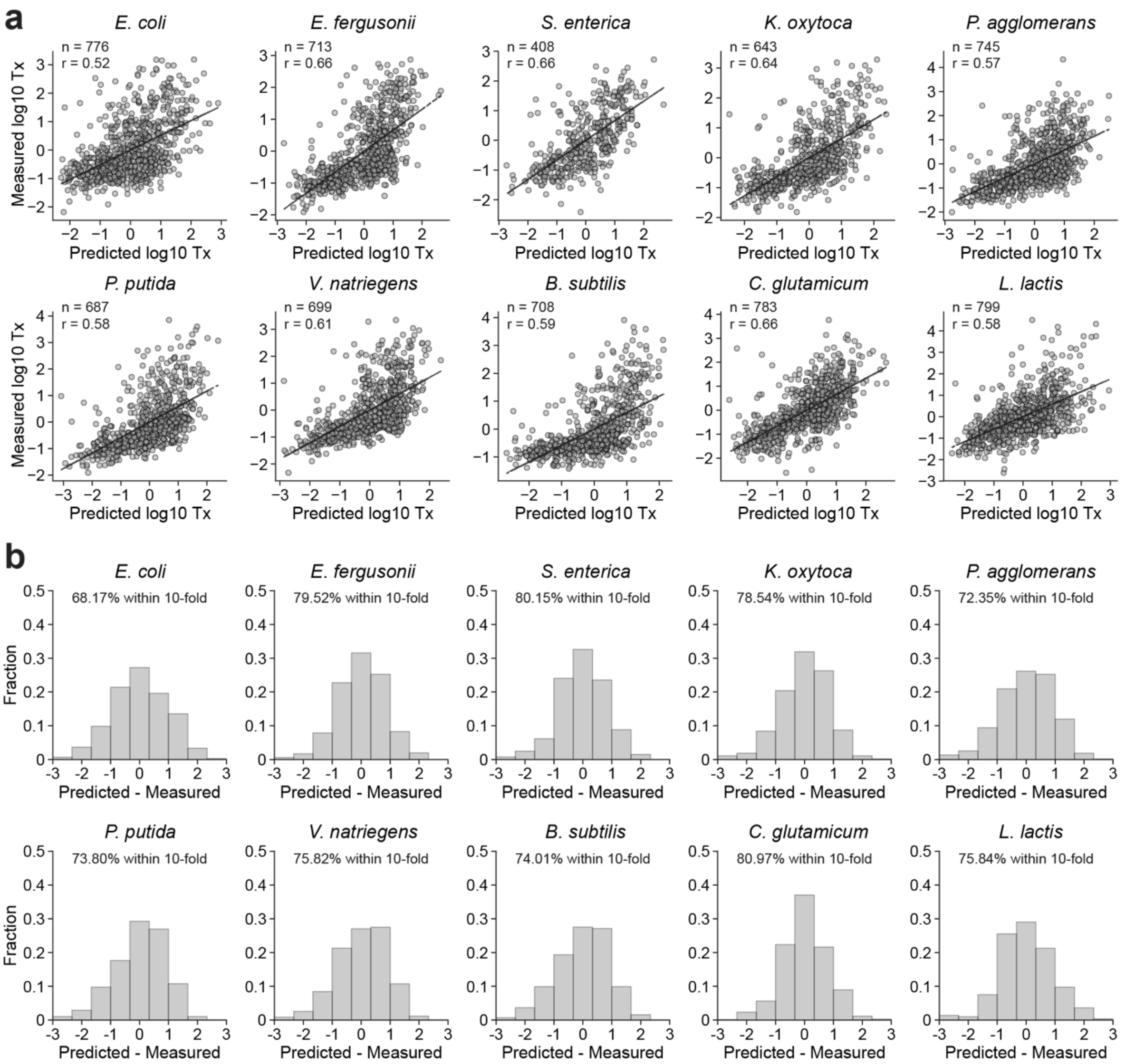

Appendix Figure S11. Evaluation of linear regression models on a separate dataset. (a) The linear regression models, trained on the RS7003 library data, were tested on a separate data generated from the RS1383 library. **(b)** Distributions of prediction errors (Predicted – Measured transcription activities in $\log_{10}$ scale) on the RS1383 library data. Proportion of prediction that are within 10-fold error window is shown in each plot.

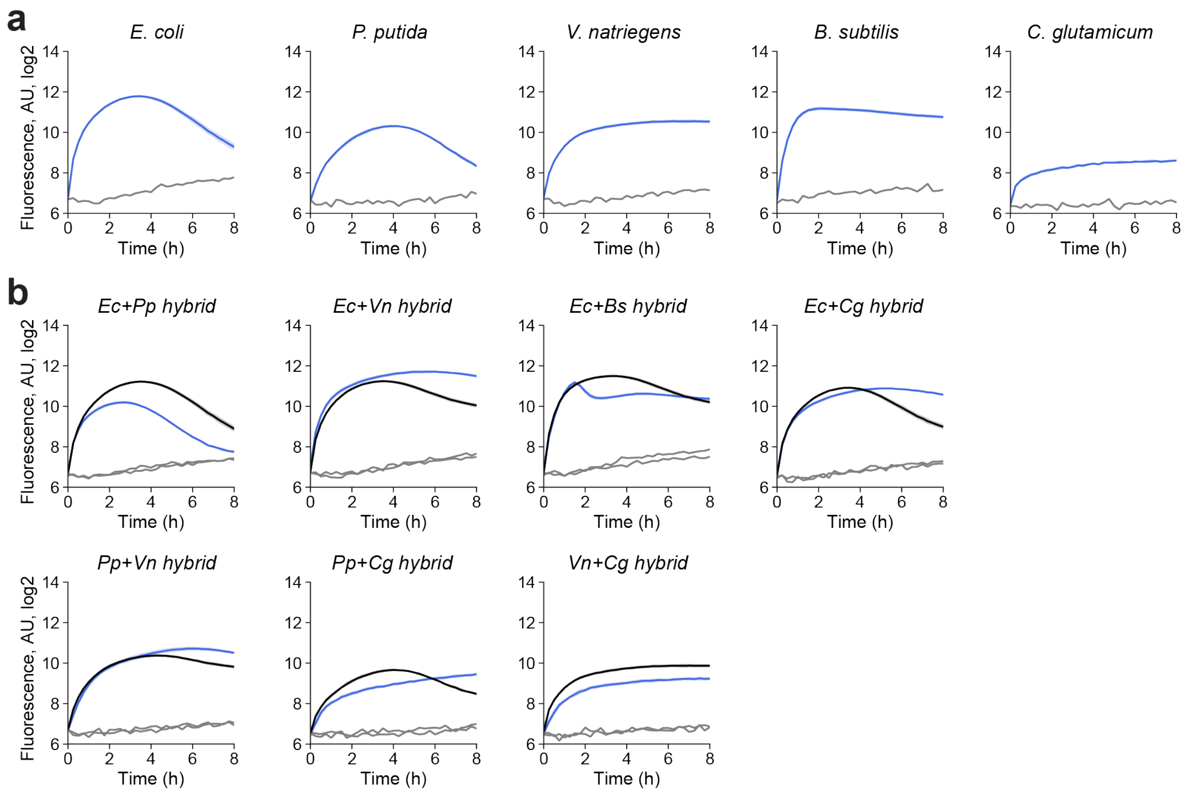

Appendix Figure S12. Transcriptional activities from dual-species hybrid lysates. (a) Time course profile of Broccoli transcription in 5 single-species source lysates. **(b)** Time course profile of Broccoli transcription in 7 dual-species hybrid lysates. Blue: Observed Broccoli intensity, Grey: Negative control without DNA template, Black: Broccoli intensity predicted by an additive model summing Broccoli intensities from constituent single-species lysates. For Broccoli expression, 25 nM of pTOPO-F30-Broccoli plasmid was used as a template. Shaded regions represent standard deviation of three biological replicates.

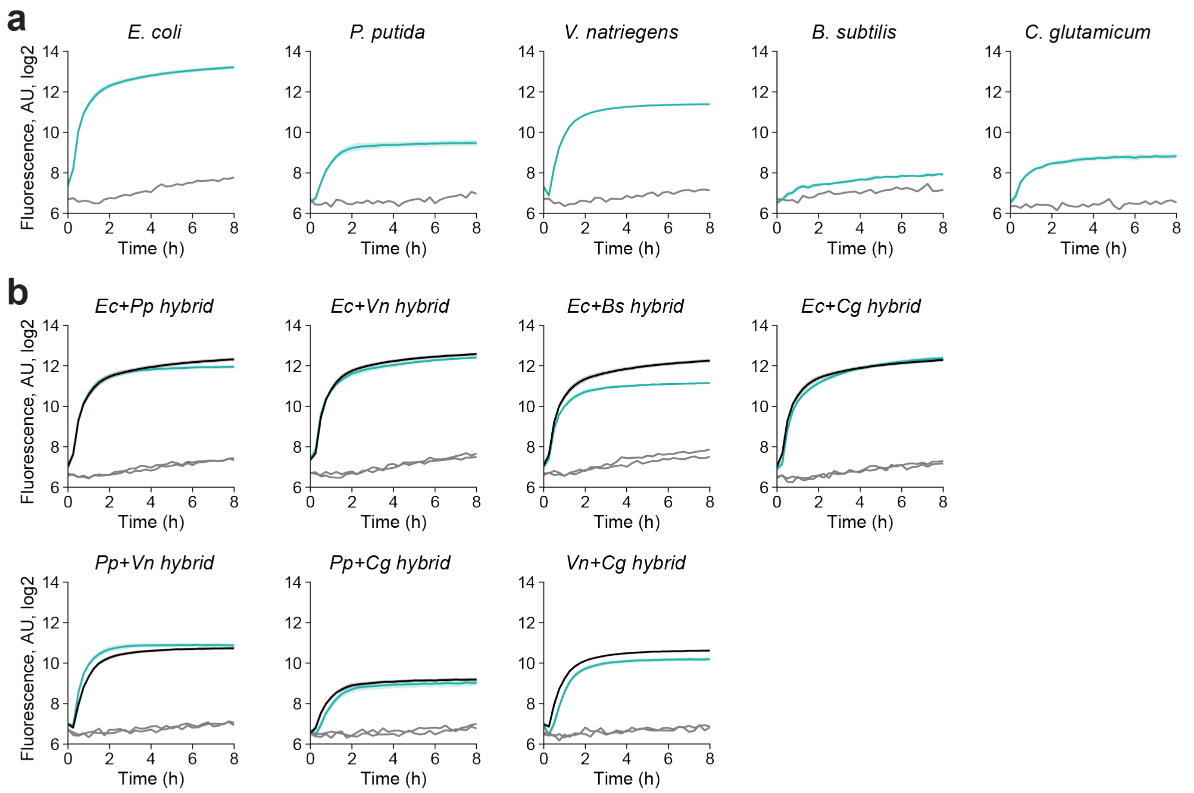

Appendix Figure S13. Translational activities from dual-species hybrid lysates. (a) Time course profile of GFP translation in 5 single-species source lysates. **(b)** Time course profile of GFP translation in 7 dual-species hybrid lysates. Green: Observed GFP intensity, Grey: Negative control without DNA template, Black: GFP intensity predicted by an additive model summing GFP intensities from constituent single-species lysates. For GFP expression, 25 nM of pTXTL-P70a-deGFP plasmid was used as a template. Shaded regions represent standard deviation of three biological replicates.

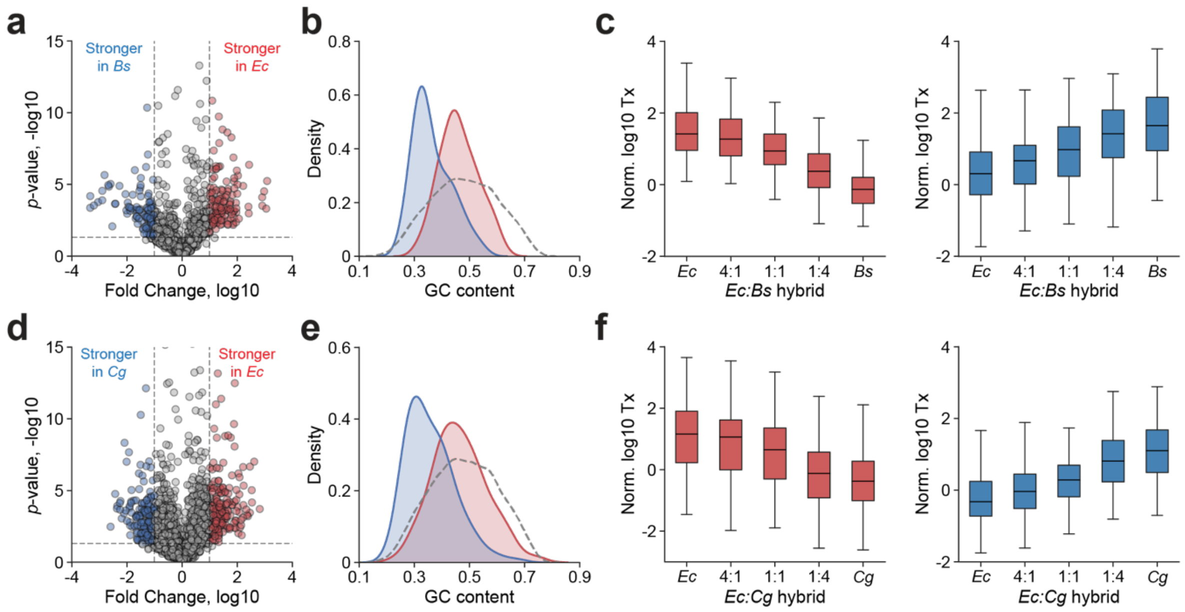

Appendix Figure S14. Hybrid lysates alleviate species-selectivity of promoters. (a) Volcano plot for relative changes of transcription measurements in original *E. coli versus B. subtilis* lysates. Promoters that are >10-fold stronger in *E. coli* (red) or *B. subtilis* (blue) with statistical significance (*p*-value < 0.05, Student's t-test) were selected for further analysis. **(b)** GC content distributions of the *E. coli*-selective (red) and *B. subtilis*-selective (blue) promoters. GC content distribution of all promoters is also shown as a gray dashed line. **(c)** Box plots of transcriptional activity distributions of the *E. coli*-selective (red) and *B. subtilis*-selective (blue) promoters in original and hybrid lysates with different mixing ratios. **(d)** Volcano plot for relative changes of transcription measurements in original *E. coli versus C. glutamicum* lysates. Promoters that are >10-fold stronger in *E. coli* (red) or *C. glutamicum* (blue) with statistical significance (*p*-value < 0.05, Student's t-test) were selected for further analysis. **(e)** GC content distributions of the *E. coli*-selective (red) and *C. glutamicum*-selective (blue) promoters. GC content distribution of all promoters is also shown as a gray dashed line. **(f)** Box plots of transcriptional activity distributions of the *E. coli*-selective (red) and *C. glutamicum*-selective (blue) promoters in original and hybrid lysates with different mixing ratios. Box plots are displaying the interquartile range (IQR) with median values (black line) and whiskers extending to the highest and lowest points within 1.5x the IQR. For normalization, transcription levels in $\log_{10}$ scale were transformed to Z-score. All measurements are based on two biological replicates.
